# Effect of Waterjet Machining Parameters on the Cut Quality of PP and PVC-U Materials Coated with Polyurethane and Acrylate Coatings

**DOI:** 10.3390/ma14247542

**Published:** 2021-12-08

**Authors:** Miroslav Müller, Viktor Kolář, Jan Šulc, Rajesh Kumar Mishra, Monika Hromasová, Bijoya Kumar Behera

**Affiliations:** 1 Department of Material Science and Manufacturing Technology, Faculty of Engineering, Czech University of Life Sciences Prague, Kamycka 129, 165 00 Prague, Czech Republic; muller@tf.czu.cz (M.M.); vkolar@tf.czu.cz (V.K.); honzasulc31@gmail.com (J.Š.); 2 Department of Electrical Engineering and Automation, Faculty of Engineering, Czech University of Life Sciences Prague, Kamycka 129, 165 00 Prague, Czech Republic; hromasova@tf.czu.cz; 3 Department of Textile & Fiber Engineering, Indian Institute of Technology Delhi, New Delhi 110016, India; behera@textile.iitd.ac.in

**Keywords:** waterjet technology, machining, polymer, surface treatment, SEM, cut quality

## Abstract

The article focuses on the machining of polymeric materials polypropylene (PP) and un-plasticized poly vinyl chloride (PVC-U) after surface treatment with polyurethane and acrylate coatings using waterjet technology. Two types of waterjet technologies, abrasive waterjet (AWJ) and waterjet without abrasive (WJ), were used. The kerf width and its taper angle, at the inlet and outlet of the waterjet from the workpiece, were evaluated. Significant differences between AWJ and WJ technology were found. WJ technology proved to be less effective due to the creation of a nonuniform cutting gap and significant burrs. AWJ technology was shown to be more efficient, i.e., more uniform cuts were achieved compared to WJ technology, especially at a cutting head traverse speed of 50 mm·min^−1^. The most uniform kerf width or taper angle was achieved for PP + MOBIHEL (0.09°). The materials (PP and PVC-U) with the POLURAN coating had higher values of the taper angle of the cutting gap than the material with the MOBIHEL coating at all cutting head traverse speeds. The SEM results showed that the inappropriate cutting head traverse speed and the associated WJ technology resulted in significant destruction of the material to be cut on the underside of the cut. Delamination of the POLURAN and MOBIHEL coatings from the base material PP and PVC-U was not demonstrated by SEM analysis over the range of cutting head traverse speeds, i.e., 50 to 1000 mm·min^−1^.

## 1. Introduction

In the automotive industry, as well as in other sectors, coated plastic materials are increasingly replacing conventional metal materials. Plastic products are distinguished by their low weight, toughness, sufficient strength, and, as a rule, not very high price [1]. The surface finish of plastic products often gives the impression of higher quality. Surface treatment is carried out on already finished semifinished products. In many cases, however, it is carried out on flat blanks which are subsequently modified to the desired shape and dimensions by various methods of cutting the material [2]. Plastics are inherently hydrophobic materials with low surface energy and, therefore, do not adhere well to other materials that come into contact with them [1]. For this reason, the condition that the surface tension of the coating must be lower than the critical surface tension of the plastic material must be observed [1]. In the case where the critical surface tension of the plastic is too low to achieve the required wetting and adhesion properties, it must be increased by means of appropriate pretreatments [3].

When using conventional machining methods for composite materials and coated plastics, fiber pullout, delamination, and fiber breakage can occur, particularly due to tool-to-workpiece contact [4]. An alternative is the promising nonconventional method of machining industrial components, i.e., waterjet cutting [5,6].

However, even with this alternative method using a water jet, the fact that negative aspects similar to conventional machining methods can occur if the cutting process is not set up correctly must be taken into account [7].

The waterjet technology is characterized by a high flexibility of setup machining parameters, which can be used especially in the machining of polymer composite materials, which are characterized by considerable variability [8]. As early as 1999, Wang focused on machining of polymer-based composite materials and performed 64 different tests to evaluate the effect of level of jet angles, level of water pressures, traverse speed, etc [8]. The results showed the importance of optimizing the cutting process leading to efficient machining of polymer based materials. Shanmungan et al. [9] mainly focused on the research of traverse speed, which is an important factor affecting the quality of cut and the associated delamination of each layer.

The research showed that a traverse speed of 750 mm·min^−1^ does not provide the waterjet with sufficient kinetic and erosive ability to produce a cut across the entire cross-section. Delamination can occur in composites and layered materials, and there is a large variance in results of up to 230%. The above conclusion was also demonstrated by electron microscopy findings [2].

The waterjet cutting technology is, therefore, based on the principle of extruding the water jet under high pressure. This secondarily accelerates the water jet to a high speed. In the mixing chamber, the passing waterjet creates an underpressure and, thus, carries air with it. Due to this underpressure, the abrasive medium is drawn into the two-phase jet, creating a three-phase flow. This three-phase jet concentrates on the focusing tube and increases the cutting effect even at faster traverse speeds [10,11].

Often, the addition of abrasive particles or the pulsating effect of the liquid is used to increase the effect [2]. The addition of abrasive particles or the use of a pulsating water jet increases the effect of the water jet technology [10,11]. This is a promising technology for the separation of heterogeneous materials.

The biggest problem in using waterjet technology is the heterogeneous quality of the machined surface. This heterogeneity is manifested by different cutting quality parameters. These parameters include surface roughness, deviation of the machined surface from the vertical cutting plane, delamination, and, last but not least, the characteristic appearance of the cutting surface, i.e., curved lines on the machined surface [12,13]. All these phenomena significantly affect the use of this technology and must be addressed when setting the optimum conditions for the production process. Damage to composite materials and polymers due to dynamic loading is significant. It causes delamination which may not be apparent by visual inspection [14] and is a major problem in machining [6]. Delamination is caused by water wedging and shock waves in the initial phase, as well as by the deposition of abrasive particles at the interface of two different materials [2,6]. There are several studies that dealt with the analysis of the effect of input parameters on the quality of cut, delamination, etc. to optimize the process parameters for cutting a given material using waterjet technology [6]. The studies mainly dealt with three main factors, i.e., cutting head traverse speed, amount and type of abrasive, and pressure [6].

There is a growing demand for materials that have to fulfil specific functions and consist of multiple components. For these materials, research potential arises in relation to their machining process. Due to the variability of these materials, there is no exact general solution [15]. Various factors are taken into account in relation to the acceptability of the machining process, which depend on the setup parameters and the physical properties of the chosen material.

This paper presents a research study focused on erosive machining of polymeric materials with surface finish, which were prepared on the basis of a request from a manufacturing company.

In this study, abrasive waterjet (AWJ) and waterjet without abrasive (WJ) machining techniques were used to machine PP and PVC-U materials coated with polyurethane and acrylate. The main objective of this study was to investigate the quality of the cutting gaps of the tested materials and, on the basis of the test results, to determine the required optimum machining parameters, i.e., the results with respect to the desired output of a quality cut showing no damage at the interface of the plastic and coatings while maintaining the uniformity of the cut, i.e., at the inlet and outlet.

Qualitative parameters were evaluated on the basis of the profile and section geometry of the tested materials. SEM analysis was performed to study the cutting process and cutting mechanisms affecting the specific materials tested.

## 2. Materials and Methods

For the experimental part, four samples of two types of materials were used, on which layers of acrylic or polyurethane coatings were applied. The test materials were first machined into test specimens with dimensions of 210 × 150 × 4 mm. Subsequently, their surface was mechanically treated, a plastic primer was applied, and two types of organic coatings were applied.

### 2.1. Materials

**Figure 1:**
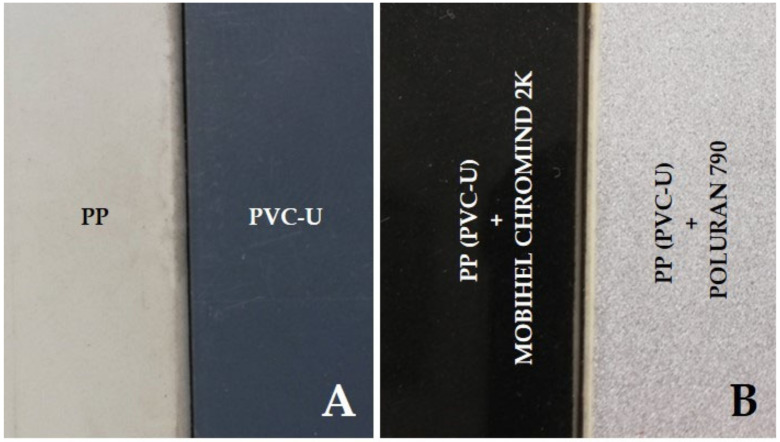
Test samples: (**A**) polymers before coating; (**B**) polymers after coating.

The following polymers were chosen for the experimental part and are shown in Figure 1A:

- unplasticized polyvinyl chloride (PVC-U),
- polypropylene (PP).

The test specimens were first cut with a band saw into 210 × 150 × 4 mm plates. Subsequently, the surface of the specimens was ground with an eccentric grinder using P400 and P800 grit grinding wheels. The sanding step was performed to remove coarse dirt, as well as distort and roughen the surface of the material, which helps to improve the adhesion of the coatings. The surface was then cleaned with a silicone cleaner to remove grease and other chemicals from the surface that would hinder the application and anchorage of the coatings. In the next step, a plastic primer was applied to the surface of the boards. The purpose of the plastic primer was to etch the surface of the plastic and create an intermediate layer that would improve the adhesion and wettability of the subsequent coatings. After the plastic primer weathered, the organic coatings were applied. The coatings were applied by pneumatic spray technology. After two coats were applied 15 min apart, the coating was cured at 20 °C for 24 h. Two types of coatings were chosen for spraying (see Figure 1B):

- Polyurethane coating Poluran 790, shade RAL 9006 (marked as POLURAN).
- Acrylic coating Mobihel Chromind 2K, shade RAL 9005 (marked as MOBIHEL).

### 2.2. Methods

**Figure 2:**
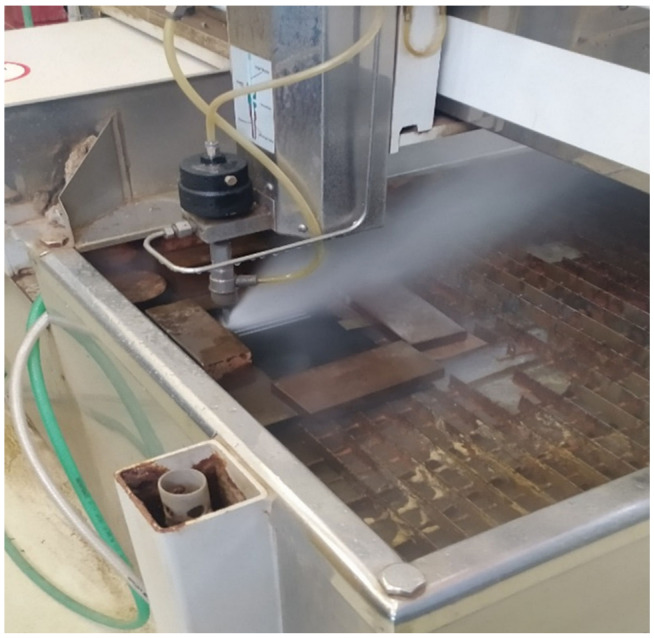
AWAC CNC waterjet cutting machine AWJ CT 0806: waterjet cutting process.

The test cuts were made using a water jet on AWAC CNC AWJ CT 0806 (AWAC, s. r. o., Prague, Czech Republic) (see Figure 2).

The research used previous experience in machining experimentally developed polymer composites with natural and synthetic reinforcement produced by vacuum infusion and testing of functional surfaces on polymer and metal materials.

**Figure 3:**
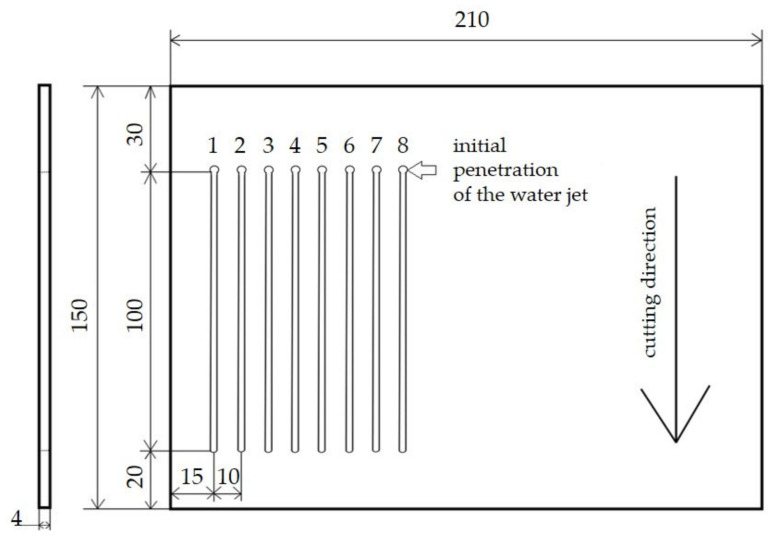
Layout of the waterjet cuts on the test specimen.

Eight cuts were made on each test specimen, as shown in Figure 3. Two types of waterjet were used. One was a pure WJ waterjet (no abrasive added), while the other was an abrasive AWJ waterjet. The abrasive added to the waterjet for the AWJ technology was Australian GARNET MESH 80, which is recommended by the equipment manufacturer. The abrasive was added into the waterjet by the Bimba Flat at an intensity of 439 ± 70 g × min^−1^.

The variable technological parameters for each cut are shown in Table 1. These values were used for all samples.

The following parameters were constant:

- working water pressure: 380 MPa;
- nozzle diameter: 0.8 mm;
- distance of the nozzle above the material to be cut: 3 mm;
- angle of the nozzle above the material to be cut: 90°;
- abrasive mass flow: 439 ± 70 g·min^−1^;
- the type and size of the abrasive: Garnet, MESH 80.

In the experimental part, the gaps on the coated polymer materials were evaluated under a stereoscopic microscope, and the width of the cutting gap at the inlet and outlet of the waterjet from the material was evaluated. The value of the taper angle of the cutting gap was calculated as a function of the waterjet cutting parameter setting.

**Figure 4:**
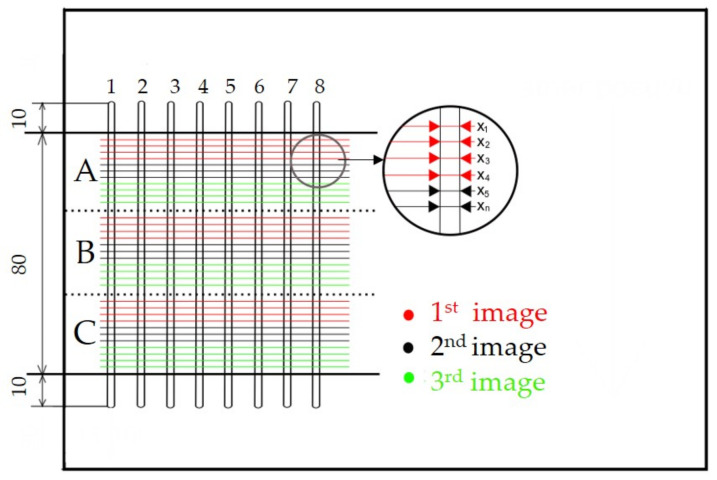
Procedure for measuring the kerf width of the cutting gap.

The kerf width was evaluated at the inlet and outlet of the water jet, i.e., at the top and bottom of the material. The measured area of the sample was located approximately 10 mm from the beginning of the kerf (initial penetration of the water jet) and 10 mm from the end of the kerf. The reason for shifting the start and end of the measurement was to eliminate biased results caused by the rise and fall time of the cutting head, i.e., the moment before the cutting head reaches the desired cutting speed. A working area was delineated on each sample and divided into three parts—A, B, and C (see Figure 4). For each cut, three images were taken in each part (A, B, C), for a total of nine images. In each image, 3–4 values were measured. In total, 11 values were measured in each section, i.e., 33 values for one section. On the side of the water jet outlet from the workpiece, only cuts that showed a regular shape without significant burrs were evaluated, i.e., only for the AWJ technology. The WJ cut showed an irregular shape and significant burrs for all types of materials tested. The measurement of the kerf width was performed on a Zeiss Stemi 508 (Carl Zeiss, s. r. o, Prague, Czech Republic) stereoscopic microscope with an Axiocam (Carl Zeiss, s. r. o, Prague, Czech Republic) digital camera, as shown in Figure 4.

**Figure 5:**
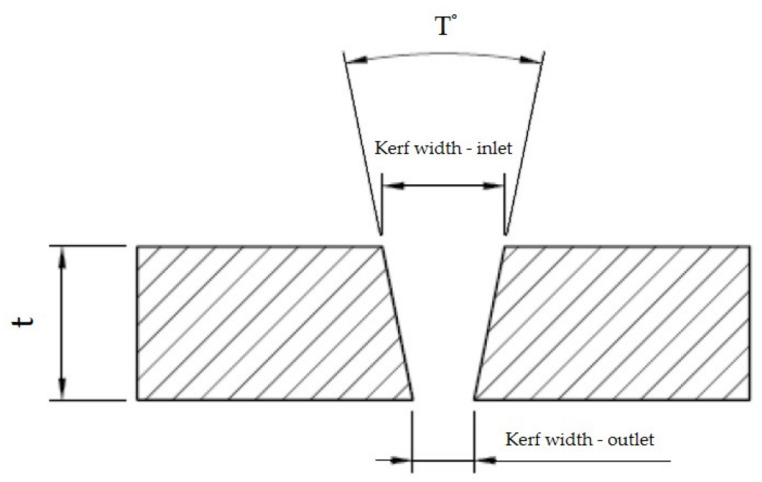
The principle of measuring the width of the cutting gap at the inlet and outlet of the water jet from the workpiece.

The taper angle “T°” of the cutting gap was calculated on the basis of the measured values of the width of the cutting gap at the inlet and outlet of the waterjet from the material (Figure 5) for each specific gap according to Equation (1) [16,17,18]. The thickness “t” of all tested materials was constant, i.e., 4 mm. The taper angle of the cutting gap was evaluated only for the gaps formed by AWJ technology. This angle could not be evaluated for the gaps formed by WJ technology, because for none of these gaps could the width be measured at the waterjet outlet of the material, since the formed gaps showed a significantly irregular shape on this side.

$$T{}^{\circ}=arctg\left(\frac{kerf\;width_{inlet}-kerf\;width_{outlet}}{2\times t}\right).$$

The cross-sectional area was evaluated by SEM (scanning electron microscopy) on a TESCAN MIRA 3 GM (Tescan Brno s.r.o., Brno, Czech Republic) microscope and a Quorum Q150R ES (Tescan Brno s.r.o., Brno, Czech Republic)—sputtering deposition rate using gold.

All measured data, i.e., the width of the cutting gaps created by the WJ and AWJ technologies, were statistically tested using the STATISTICA 14 (version 14, StatSoft CR, Prague, Czech Republic) program using the ANOVA (Analysis of Variance) F-test at the significance level α = 0.05, i.e., the hypothesis H0 presents a statistically insignificant difference between all measured data (*p* > 0.05), and the alternative hypothesis H1 presents a rejection of the hypothesis H0, i.e., that there is a statistically significant difference between all measured data (*p* < 0.05). The *p*-value represents the lowest significance level of the test at which we rejected the hypothesis.

## 3. Results and Discussion

**Figure 6:**
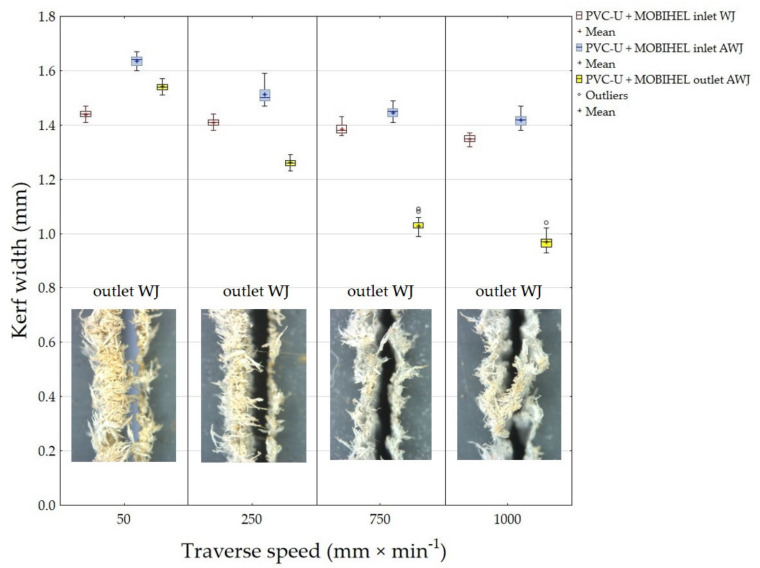
Dependence of the kerf width on the cutting head traverse speed and waterjet type for PVC-U + MOBIHEL.

**Figure 7:**
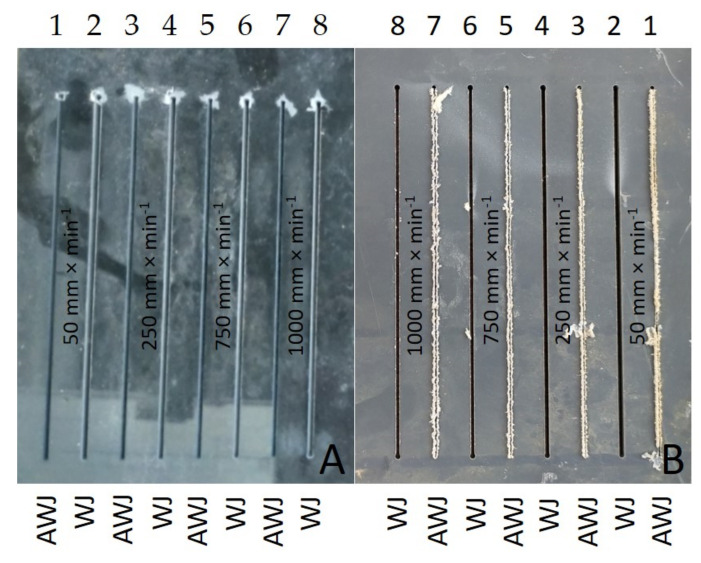
Cutting gaps in PVC-U + MOBIHEL: (**A**) WJ and AWJ inlet; (**B**) WJ and AWJ outlet.

Figure 6 presents the results of the width of the cutting gap at the inlet and outlet of the waterjet of PVC-U + MOBIHEL. When using WJ (water jet without added abrasive), the width of the cutting gap on the inlet side of the waterjet showed a gradually decreasing trend depending on the increasing traverse speed of the cutting head. The difference between the average value of the width of the cutting gap on the inlet side of the waterjet at traverse speeds of 50 mm·min^−1^ and 1000 mm·min^−1^ was only 0.09 mm. The width of the cutting gap on the outlet side of the WJ could not be measured because this side of the workpiece exhibited a highly irregular shape, which was caused by abrasion of the material being cut along the cutting gap (see Figure 7; cuts 1, 3, 5, and 7). The difference in the average value of the cutting gap width between the traverse speeds of 50 mm·min^−1^ and 1000 mm·min^−1^ at the AWJ inlet reached a value of 0.22 mm. At the waterjet outlet of the material, the difference between the cutting gap widths at different traverse speeds was even more noticeable. The difference in the average value of the cutting gap width at the waterjet outlet at traverse speeds of 50 mm·min^−1^ and 1000 mm·min^−1^ was 0.57 mm. From Figure 6, it can be seen that the difference in the cutting gap width between the inlet and outlet of the waterjet increased with increasing traverse speed of the cutting head in AWJ technology.

As the traveling speed of the cutting head in AWJ technology increased, the coefficient of variation of the measured values for the width of the cutting gap at the outlet of the waterjet from the machined material increased. At a cutting head traverse speed of 50 mm·min^−1^, the coefficient of variation was 0.93%. At a speed of 1000 mm·min^−1^, the coefficient of variation was 2.41%. These results are consistent with the increasing unevenness of the cutting gap at higher cutting head traverse speeds. Similar results were obtained for the other experimental variations.

If the cutting gap width at the outlet of the waterjet from the material is smaller than at the inlet, it is obvious that the waterjet loses kinetic energy when passing through the material to be cut. Insufficient removal of the material to be cut results from the loss of kinetic energy as the waterjet passes through [8].

Figure 7A,B show the inlet and outlet sides of the WJ and AWJ in the PVC-U + MOBIHEL material. Figure 7A shows the inlet of the AWJ and WJ into the material. The inlet side of the waterjet into the material shows a uniform cutting gap without significant burrs. Figure 7B shows the outlet of the waterjet from the material; in particular, for the WJ technology, a nonuniform cutting gap with significant burrs was observed. Similar results were obtained for all types of materials tested (see Figures 9, 12, and 14). Hejjaji et al. [19] demonstrated the significance of the effect of the waterjet cutting head traverse speed in machining polymeric materials and also clearly demonstrated the significance of the cutting head traverse speed when using both AWJ and WJ technologies, i.e., the significance of its setting and its effect on the overall quality of the cutting surface.

**Figure 8:**
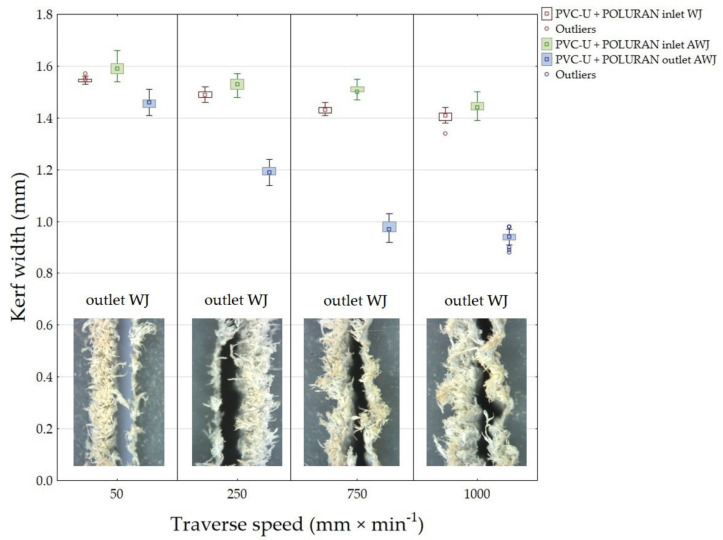
Dependence of the width of the cutting gap on the traverse speed of the cutting head and the type of water jet for PVC-U + POLURAN.

Figure 8 shows the results of the measured width of the cutting gap at the inlet and outlet of the water jet for PVC-U + POLURAN. When cutting WJ, the width of the cutting gap on the inlet side of the waterjet into the material showed a decreasing trend with gradually increasing traverse speed of the cutting head. The difference between the average value of the width of the cutting gap on the inlet side of the waterjet when cutting with WJ and traverse speeds of 50 mm·min^−1^ and 1000 mm·min^−1^ was 0.14 mm. The width of the cutting gap on the outlet side from the material could not be measured again because the said side of the workpiece showed a significantly irregular shape, which was caused by a thick layer of burrs of the cut material along the edge of the cutting gap. The difference in the average value of the width of the cutting gap between traverse speeds of 50 mm·min^−1^ and 1000 mm·min^−1^ at the AWJ inlet to the material was 0.15 mm. At the AWJ outlet from the material, the decreasing trend of the cutting gap width was even more evident, especially between cutting head traverse speeds of 50 mm·min^−1^ and 750 mm·min^−1^. There was no longer such a difference between the values of the cutting gap width at traverse speeds of 750 mm·min^−1^ and 1000 mm·min^−1^. The difference in the average value of the cutting gap width at the waterjet outlet from the material at traverse speeds of 50 mm·min^−1^ and 1000 mm·min^−1^ was 0.51 mm.

**Figure 9:**
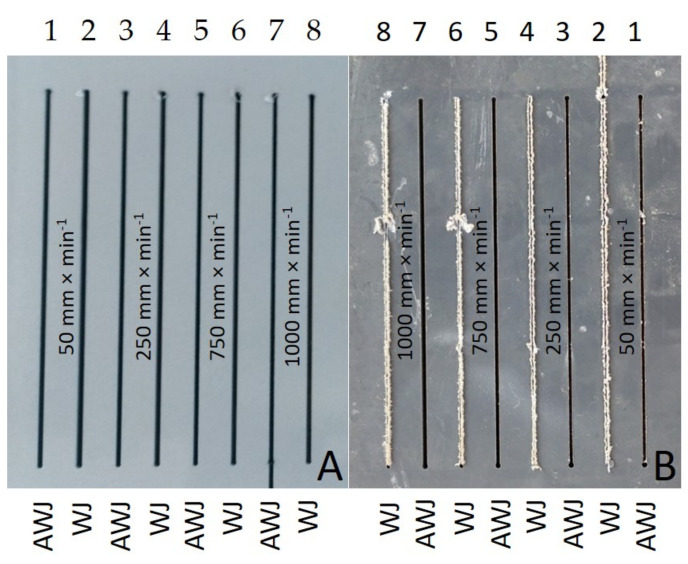
Cutting gaps in PVC-U + POLURAN: (**A**) WJ and AWJ inlet; (**B**) WJ and AWJ outlet.

Figure 9 shows the inlet and outlet sides of the WJ and AWJ in PVC-U + POLURAN.

**Figure 10:**
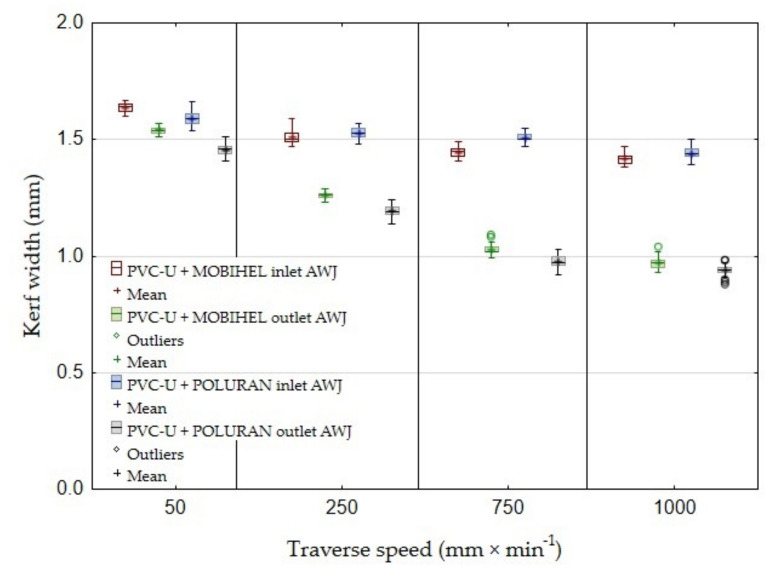
Comparison of the width of cutting gaps created by AWJ technology on the inlet and outlet side of the waterjet in PVC-U material with different surface treatments.

Figure 10 shows a comparison of the values of the width of the cutting gaps created by the AWJ technology at the inlet and outlet of the water jet as a function of the cutting head traverse speeds for the same base materials with different surface treatments, i.e., PVC-U + MOBIHEL and PVC-U + POLURAN. A comparison of the AWJ inlet cutting gap widths shows that, for PVC-U + MOBIHEL, the inlet cutting gap widths at cutting head traverse speeds of 250, 750, and 1000 mm·min^−1^ were lower than the cutting gap widths for PVC-U + POLURAN. An exception occurred at a cutting head traverse speed of 50 mm·min^−1^ when the gap width of PVC-U + MOBIHEL was higher. As the cutting head traverse speed increased, the difference between the width of the cutting gap in the material at the inlet and outlet of the AWJ increased. As can be seen from Figure 10, the most uniform cutting gap width between the inlet and outlet of the AWJ was achieved at a cutting head traverse speed of 50 mm·min^−1^ for the PVC + MOBIHEL material, when the difference between the inlet and outlet was 0.1 mm.

**Figure 11:**
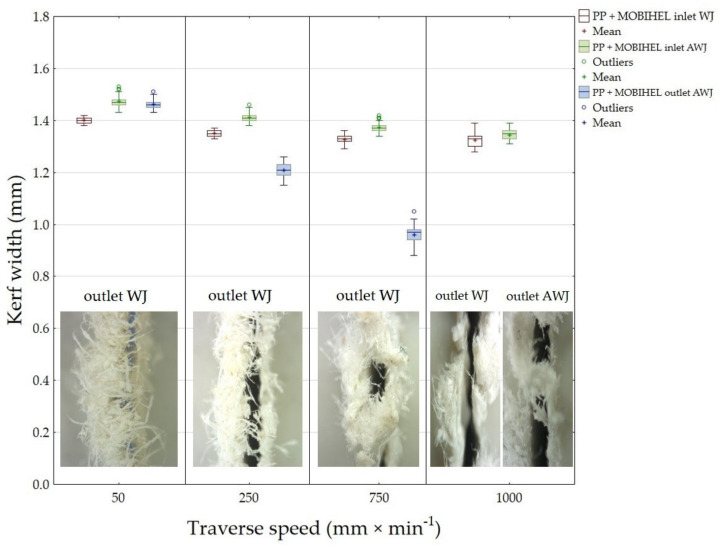
Dependence of the cutting gap width on the cutting head traverse speed and waterjet type for PP + MOBIHEL.

**Figure 12:**
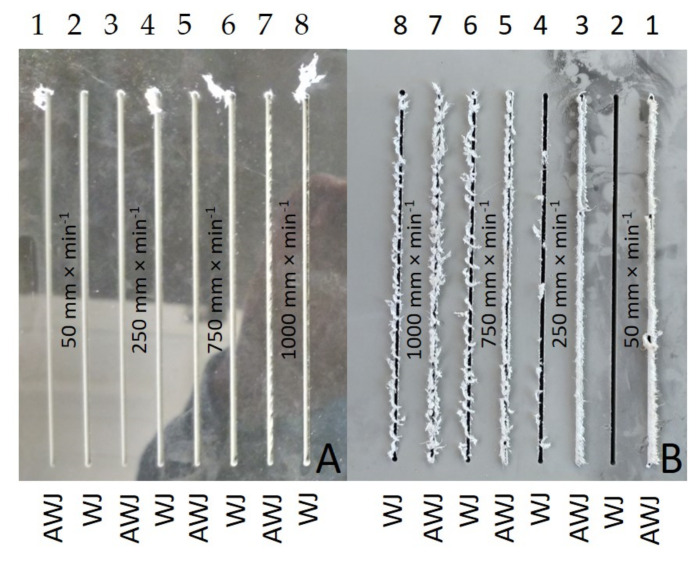
Cutting gaps in PP + MOBIHEL: (**A**) WJ and AWJ inlet; (**B**) WJ and AWJ outlet.

The results of the width of the cutting gap at the inlet and outlet of the water jet from PP + MOBIHEL are presented in Figure 11. The cuts produced by WJ technology showed a gradually decreasing trend on the inlet side of the material as the traverse speed of the cutting head increased. In addition, the average value of the cutting gap width at traverse speeds of 750 mm·min^−1^ and 1000 mm·min^−1^ was the same, i.e., 1.33 mm. The difference between the average value of the cutting gap width on the entry side at the extreme traverse speeds was only 0.07 mm. The width of the cutting gap created by WJ technology at the waterjet outlet was not measured, because this side of the workpiece showed a significantly irregular shape caused by the burrs of the material being cut along the cutting gap. The gaps produced by AWJ technology showed a gradually decreasing trend in gap width on the entry side of the waterjet with a gradually increasing value of the cutting head traverse speed. The difference in the average value of the width of the cutting gap on the inlet side of the waterjet at traverse speeds of 50 mm·min^−1^ and 1000 mm·min^−1^ reaches a value of 0.13 mm. At the outlet of the AWJ, a steeply decreasing trend of the cutting gap width with increasing value of the cutting head traverse speed can be observed compared to the previous values. It was not possible to measure the width of the cutting gap on the outlet of the AWJ from the material at a traverse speed of 1000 mm·min^−1^ as the gap formed was highly irregular in shape. The difference between the average values of the gap widths on the outlet side using the abrasive waterjet at traverse speeds of 50 mm·min^−1^ and 750 mm·min^−1^ was 0.5 mm. The image analysis of the cuts in the PP + MOBIHEL material at the inlet and outlet of the waterjet is shown in Figure 12.

An image analysis of the cuts in the PP + MOBIHEL material at the entry and exit of the waterjet is shown in Figure 12.

**Figure 13:**
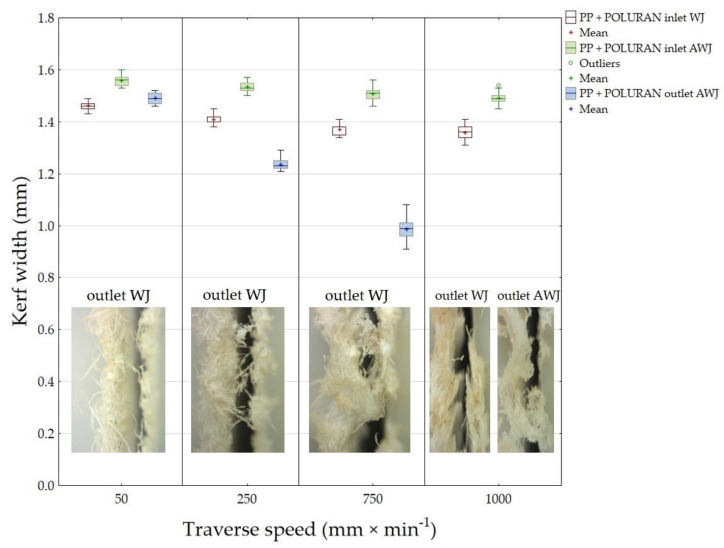
Dependence of the width of the cutting gap on the traverse speed of the cutting head and the type of water jet for PP + POLURAN.

Figure 13 presents the results of the measured width of the cutting gap at the inlet and outlet of the water jet for PP + POLURAN. The values of the cutting gap width when using WJ technology on the inlet side of the material decreased only slightly with increasing traverse speed of the cutting head. The difference in the average values of the cutting gap width between the traverse speeds of 50 mm·min^−1^ and 1000 mm·min^−1^ was 0.1 mm. The gap width at the outlet of the waterjet using WJ could not be measured again due to the significantly irregular gap edge. The width of the cutting gap using AWJ at the inlet from the material decreased even less than in the case of WJ. The difference in the average values of the width of the cutting gap at traverse speeds of 50 mm·min^−1^ and 1000 mm·min^−1^ was only 0.07 mm. In contrast, at the material outlet of the AWJ, the trend of decreasing gap width with increasing cutting head traverse speed was more pronounced. It was not possible to measure the width of the cutting gap at the outlet of the AWJ from the material at a traverse speed of 1000 mm·min^−1^ because the gap showed a significantly irregular shape caused by material abrasion along the gap edges. The average values of the gap widths at the outlet of the AWJ at the extreme traverse speeds were 0.5 mm.

**Figure 14:**
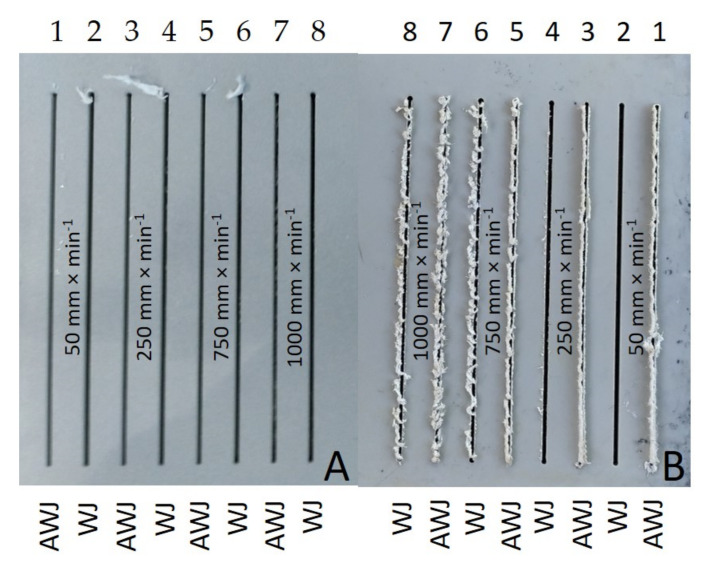
Cutting gaps in PP + POLURAN: (**A**) WJ and AWJ inlet; (**B**) WJ and AWJ outlet.

Figure 14 shows an image analysis of the inlet and outlet waterjet sections of PP + POLURAN.

**Figure 15:**
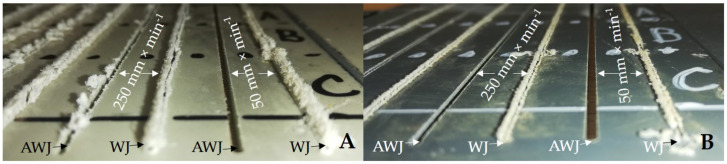
Detailed view on the outlet side of the water jet from machined materials: (**A**) PP + MOBIHEL; (**B**): PVC-U + POLURAN.

Figure 15 shows a detailed view on the outlet sides of the water jet from machined materials PP + MOBIHEL (Figure 15A) and PVC-U + POLURAN (Figure 15B). The figure shows a significant difference in the cut quality between AWJ and WJ technologies. The WJ technology exhibited a significantly irregular shape and burrs on the outlet side from the cut material, which made it impossible to measure the width of the cutting gap.

**Figure 16:**
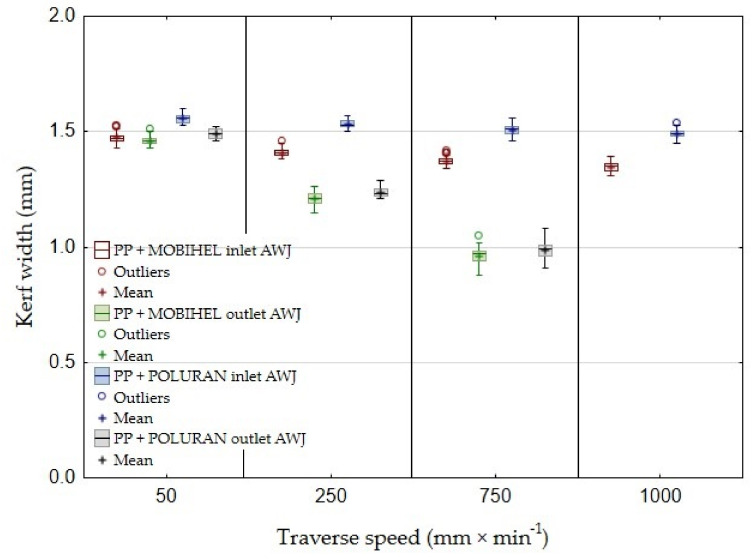
Comparison of the width of the cutting gaps created by AWJ technology on the inlet and outlet side of the waterjet in PP +POLURAN and PP + MOBIHEL.

Figure 16 shows the comparison of the width of the cutting gaps created by AWJ technology at the inlet and outlet of the water jet as a function of the cutting head traverse speeds for the same base materials with different surface treatments, i.e., PP + MOBIHEL and PP + POLURAN. A comparison of the AWJ inlet cutting gap widths shows that the PP + MOBIHEL inlet gap widths at cutting head traverse speeds of 250, 750, and 1000 mm·min^−1^ were lower than the PP + POLURAN inlet gap widths. As the cutting head traverse speed increased, the difference between the width of the cutting gap in the material at the inlet and outlet of the AWJ increased. As can be seen from Figure 16, the most uniform cutting gap width between the inlet and outlet of the AWJ was achieved at a cutting head traverse speed of 50 mm·min^−1^ for PP + MOBIHEL, where the difference between the inlet and outlet was 0.01 mm. In this case, it was the most uniform cutting gap of all the materials tested. Similar results were obtained for PVC-U + MOBIHEL.

The abovementioned results show that, in terms of the effect of cutting head traverse speed on the width of the cutting gap, the AWJ technology at a speed of 50 mm·min^−1^ was the most suitable because of the most uniform width of the cutting gap between the AWJ inlet and outlet from the material. The above conclusion shows that the water jet did not significantly lose kinetic energy when passing through the material. As the traverse speed of the cutting head increased, the loss of kinetic energy increased, which is supported by the increase in the difference in the width of the cutting gap at the inlet and outlet of the AWJ as the traverse speed of the cutting head increased. The MOBIHEL coating showed a more uniform cutting gap width on both base materials (PP and PVC-U) than the POLURAN coating.

The results of the statistical testing of the experimental variants are shown in Table 2. In terms of statistical testing of the dependence of the traverse speed of the cutting head of 50, 250, 750, and 1000 mm·min^−1^ and the WJ or AWJ waterjet type on the width of the cutting gap, it can be concluded that there was a statistically significant difference between these speed parameters at the 0.05 significance level (*p* = 0.0001). Thus, there was a strong dependence of the width of the cutting gap on the cutting head traverse speed when using both WJ and AWJ technology.

**Figure 17:**
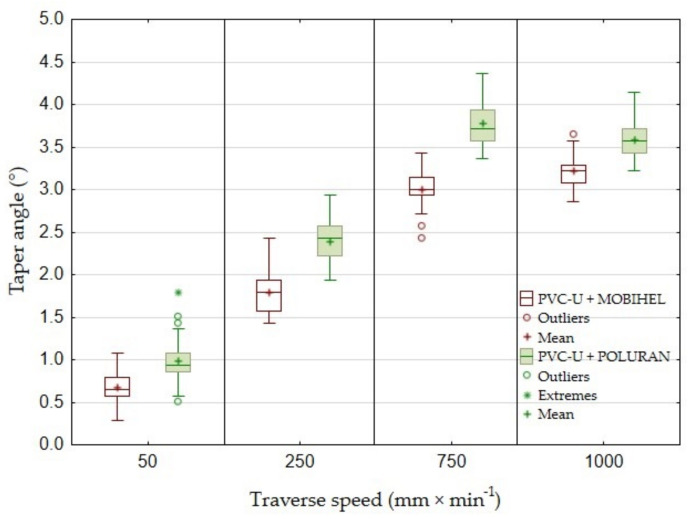
Comparison of the taper angle of the cutting gap of PVC-U + MOBIHEL and PVC-U + POLURAN.

Figure 17 shows a graph of the dependence of the taper angle of the cutting gap on the traverse speed of the cutting head when using AWJ for PVC-U + MOBIHEL and PVC-U + POLURAN. For PVC-U + MOBIHEL, it can be observed that the taper angle of the cutting gap increased linearly from a traverse speed of 50 mm·min^−1^ up to a traverse speed of 750 mm·min^−1^. At a traverse speed of 50 mm·min^−1^, the taper angle reached 0°67′. At a traverse speed of 750 mm·min^−1^, the taper angle reached 2°99′. At a cutting head traverse speed of 1000 mm·min^−1^, the trend of the angle increase slowed down, and the value of the taper angle at this traverse speed was 3°22′. From the calculated values of the taper angle for PVC-U + POLURAN, it can be concluded that, for a cutting speed of 50 mm·min^−1^ the value of the taper angle was 0°99′. However, as the traverse speed of the cutting head increased, the angle magnitude increased linearly up to a traverse speed of 750 mm·min^−1^, when the calculated angle reached a value of 3°78′. At a traverse speed of 1000 mm·min^−1^, however, the taper angle of the cutting gap decreased to 3°59′. When comparing the two curves showing the taper angle of the cutting gap of the same materials with different surface treatments at different traverse speeds, it can be concluded that the taper angle of the cutting gap of the POLURAN-coated material was greater than that of the MOBIHEL-coated material at all traverse speeds.

**Figure 18:**
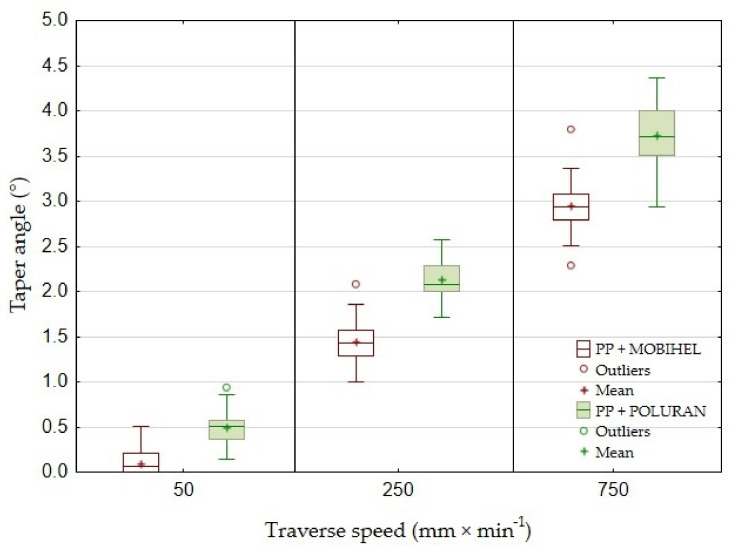
Comparison of the taper angle of the cutting gap of PP materials with different surface treatments.

The taper angle of the cutting gap depending on the traverse speed of the cutting head of PP + MOBIHEL and PP + POLURAN is shown in Figure 18. The deviation of the cutting gap surface from the perpendicularity of the ideal cut could only be determined for traverse speeds of 50, 250, and 750 mm·min^−1^ because it was not possible to measure the width of the cutting gap on the outlet side of the AWJ material at a traverse speed of 1000 mm·min^−1^. This was due to the highly irregular shape of the cutting gap. For PP + MOBIHEL, the taper angle of the gap increased almost linearly from 0°09′ at a traverse speed of 50 mm·min^−1^ to 2°95′ at a traverse speed of 750 mm·min^−1^. The taper angle curve for PP + POLURAN also increased almost linearly with increasing traverse speed. At a traverse speed of 50 mm·min^−1^ the angle became 0°49′; at a traverse speed of 750 mm·min^−1^, it reached 3°73′. When comparing the two curves for the dependence of the taper angle of the cutting gap on the traverse speed of the cutting head, it can be concluded that the taper angle of the POLURAN-coated material was higher than that of the MOBIHEL-coated material at all traverse speeds.

For greater clarity, the values of the taper angles of the cutting gap for all materials depending on the traverse speed of the cutting head are given in Table 3. Table 3 and a comparison of Figure 17 and Figure 18 show that the most uniform cutting gap taper angle was achieved at a speed of 50 mm·min^−1^ for PP + MOBIHEL, i.e., 0°09′. As the traverse speed of the cutting head increased, the taper angle of the cutting gap increased. It can also be seen that a more uniform taper angle of the cutting gap was achieved for both PP and PVC-U materials with MOBIHEL coating.

The comparison of the results presented in Table 3 and Figure 17 and Figure 18 shows the difference between different base materials, i.e., PP and PVC-U, with the same surface treatment. PVC-U + MOBIHEL showed a cutting gap taper angle of 0°09′ at a speed of 50 mm·min^−1^. PP + MOBIHEL showed an angle of 0°67′ at the same speed. It is, therefore, clear that the base material used affects the taper angle of the cutting gap. Similar results were obtained for the other variants (see Table 3 and Figure 17 and Figure 18).

Wang [8] stated that the taper angle of the cutting gap increases slightly with increasing cutting head traverse speed. The conclusion was confirmed for all types of materials tested using AWJ technology (see Table 3). The reason for the increase in the cutting gap taper angle with increasing cutting head traverse speed was the loss of kinetic energy of the waterjet during passage through the workpiece at constant abrasive dosage into the waterjet [8].

The experimental results show the effectiveness of machining polymeric materials using the AWJ method. Other results also confirmed that high-quality and efficient machining of polymeric materials can be achieved by AWJ technology [8,16].

With the correct choice of the cutting head traverse speed, the walls of the material to be cut are parallel, which means that there is no narrowing of the cut profile, and the surfaces are smooth without undulations [20]. However, if the traverse speed is high, a V-shaped cut is created. Scanning electron microscopy is one of the most useful methods for assessing surface and delamination in the materials tested [6].

**Figure 19:**
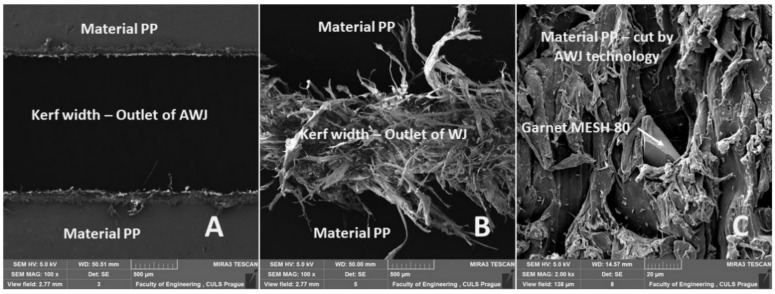
View of the cut of the tested material PP + MOBIHEL at a cutting head traverse speed of 50 mm·min^−1^: (**A**) cutting groove created by AWJ technology—outlet side of the abrasive waterjet without significant deformation of the cutting edges (MAG 100×); (**B**) cutting groove created by WJ technology—outlet side of the waterjet with significant deformation of the cutting edges (MAG 100×); (**C**) cutting area created by AWJ technology with a noticeable abrasive particle from the cutting process (MAG 2000×).

From the measurement results, a significant difference between the AWJ and WJ technology is evident from Figure 19. The difference is presented for PP + MOBIHEL material at a cutting head traverse speed of 50 mm·min^−1^. Figure 19A represents the kerf width at the exit when using AWJ technology. Figure 19A shows a regular straight cut without significant deformation and burr of PP material. Figure 19B shows an irregular cut characterized by significant burrs. From Figure 19B, it is clear that the WJ, even at a low cutting head traverse speed of 50 mm·min^−1^, did not have sufficient kinetic energy, and deformation occurred at the water jet exit. A detailed view of the cutting area using AWJ technology can be seen in Figure 19C. From Figure 19C, it can be seen that abrasive particles of Garnet MESH 80 remained in the PP material.

**Figure 20:**
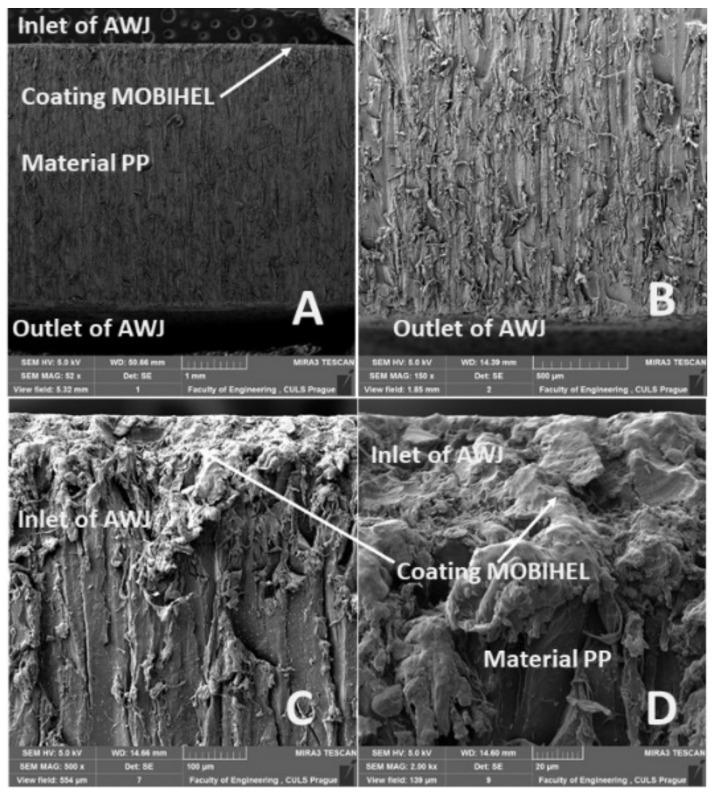
View of the cut of the tested PP + MOBIHEL, AWJ technology, cutting head traverse speed 50 mm·min^−1^: (**A**) overall cut (MAG 52×); (**B**) detailed view of the outlet of AWJ (MAG 150×); (**C**) detailed view of the inlet of AWJ (MAG 500×); (**D**) detailed view of the interface of MOBIHEL coating and PP material (2000×).

**Figure 21:**
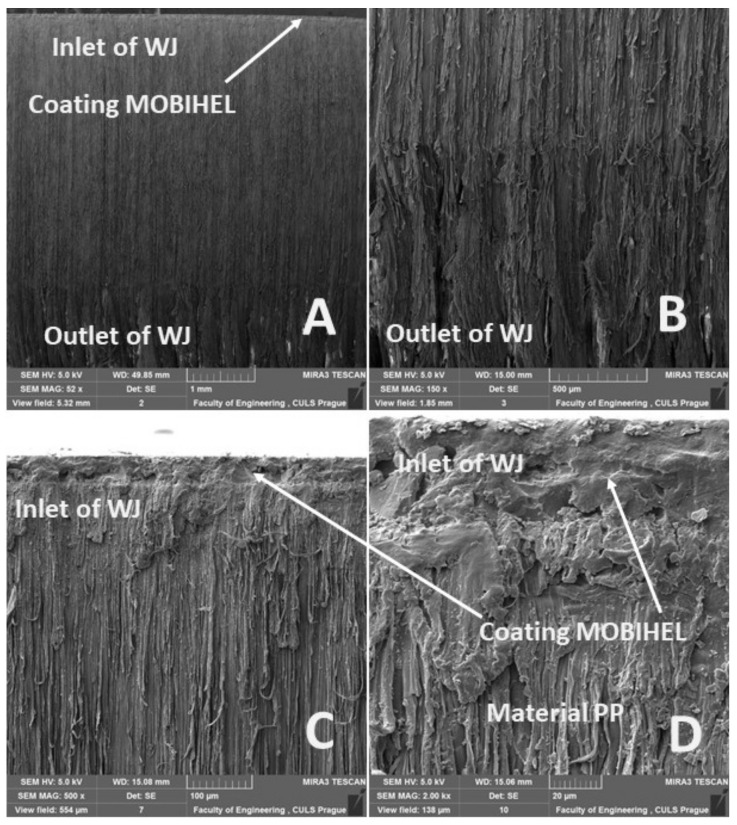
Cutting view of the tested PP + MOBIHEL, WJ technology, cutting head traverse speed 50 mm·min^−1^: (**A**) overall cut (MAG 52×); (**B**) detailed view of the outlet of WJ (MAG 150×); (**C**) detailed view of the inlet of WJ (MAG 500×); (**D**) detailed view of the interface of MOBIHEL coating and PP material (2000×).

**Figure 22:**
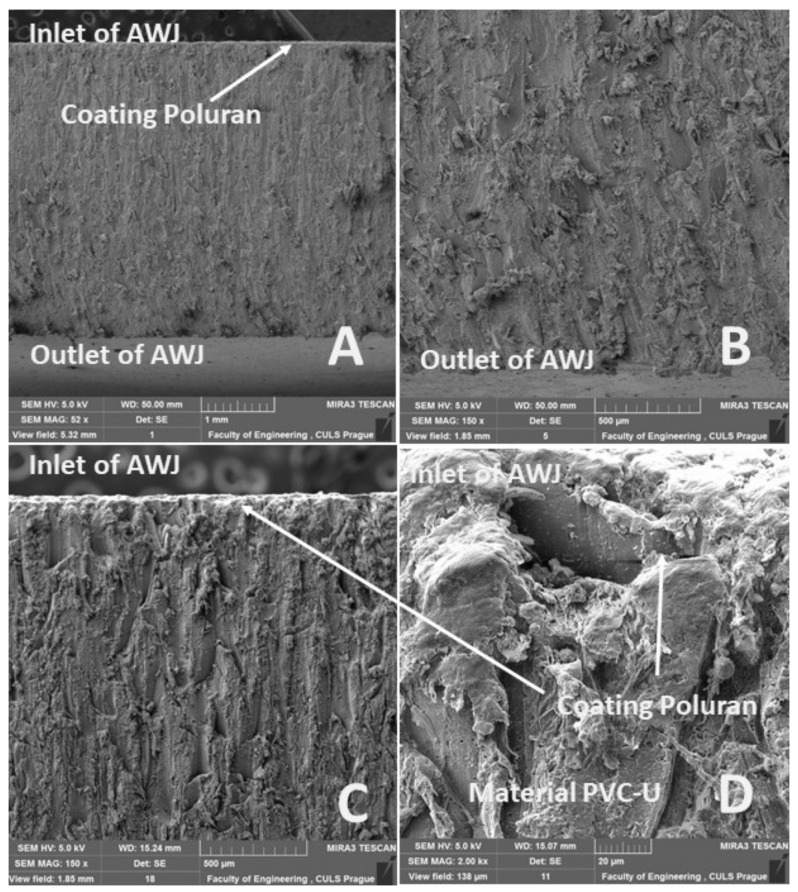
Cutting view of the tested PVC-U + POLURAN, AWJ technology, cutting head traverse speed 1000 mm·min^−1^: (**A**) overall cut (MAG 52×); (**B**) detailed view of the outlet of AWJ (MAG 150×); (**C**) detailed view of the inlet of AWJ (MAG 500×); (**D**) detailed view of the interface of POLURAN coating and PVC-U material (2000×).

**Figure 23:**
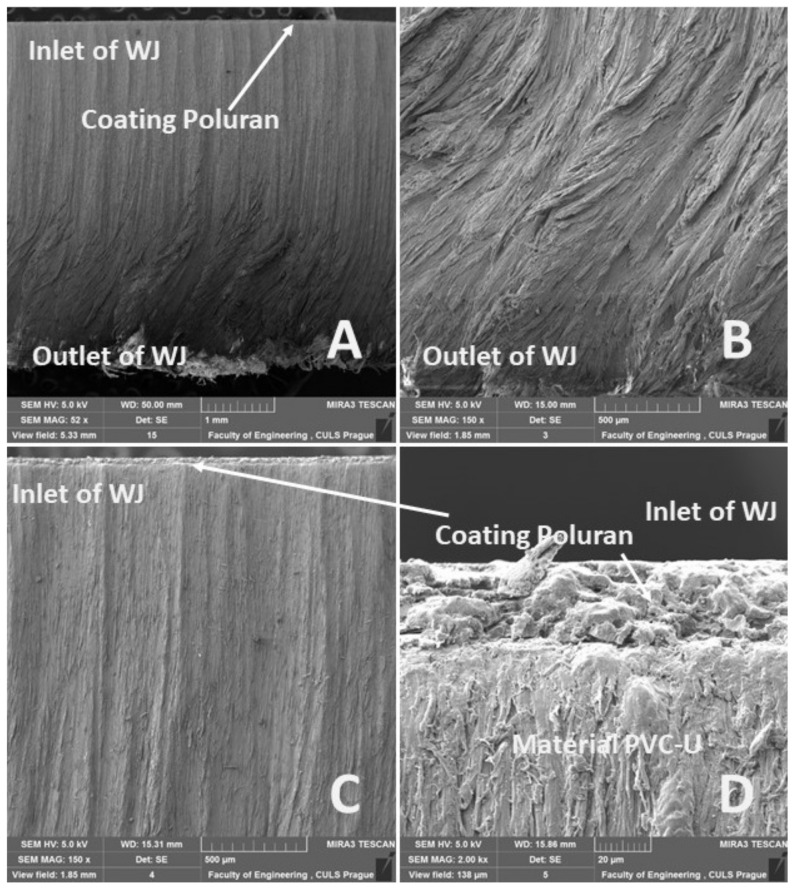
Cutting view of the tested PVC-U + POLURAN, WJ technology, cutting head traverse speed 1000 mm·min^−1^: (**A**) overall cut (MAG 52×); (**B**) detailed view of the outlet of WJ (MAG 150×); (**C**) detailed view of the inlet of WJ (MAG 500×); (**D**) detailed view of the interface of POLURAN coating and PVC-U material (2000×).

Figure 20A, Figure 21A, Figure 22A and Figure 23A show an overview view of the section area. From Figure 20A, Figure 21A and Figure 22A using AWJ technology, a more uniform texture of the cut surface is evident. From Figure 20B and Figure 22B, it can be seen that, even with different traverse speeds of the cutting head, there was no burr formation and destruction when the water jet with abrasive exited the material. Destruction and delamination can change the mechanical properties of the material after cutting [7,9]. From Figure 19A, Figure 20A,B, and Figure 22A,B, it is evident that the AWJ technology produced a better-quality, more uniform cut. This confirms the findings published by Wang [8], who stated that AWJ is an effective technology for polymeric materials, i.e., including composites. From Figure 21 and Figure 23, there was a distinct grooving highlighted at the bottom of the cut, i.e., at the point where the water jet left the cut material (Figure 21B and Figure 23B). A close look on the inlet side of the cut at different speeds is shown in Figure 20D, Figure 21D, Figure 22D and Figure 23D, where there was no delamination of the surface layer for both tested materials PP and PVC-U or for the surface treatment MOBIHEL and POLURAN (Figure 20C,D Figure 21C,D Figure 22C,D and Figure 23C,D), although it is reported in the literature that delamination of different materials can occur due to high fluid flow velocity [2,7,10], i.e., on impact.

## 4. Conclusions

The research results presented in this paper add to the knowledge of the machining process of polymeric materials with surface treatment using waterjet technology. The results on the machining of two polymeric materials, polypropylene (PP) and unplasticized polyvinyl chloride (PVC-U), after surface treatment with polyurethane and acrylic coatings shows the possibility of efficient cutting under the assumptions of the optimal cutting parameters found by the research. The WJ technology proved to be less effective due to the uneven cutting gap and significant burrs in the area of the water jet outlet from the material. The AWJ technology proved to be more effective, especially in terms of achieving a more uniform cutting gap and taper angle for both materials investigated. A significant finding is that there was no delamination of the functional view layer of the POLURAN and MOBIHEL coatings.

This is the first investigation of a polymeric material with a surface treatment that is intended for a design application. Further research, which is in preparation, will take advantage of the knowledge of the field of machining polymeric materials with other types of surface treatment to meet the desired design appearance effect. For this reason, it is secondarily possible to consider the rigor in the versatility of the tested methodology to the tested plastics with different surface treatments, which will be the focus of further research activities according to the customer’s requirement. The results found in this study can be summarized as follows:

- The measurements showed the influence of the traverse speed of the waterjet cutting head type WJ and AWJ on the width of the cutting gap. The conclusion is supported by the results presented in the graphs (Figure 6, Figure 8, Figure 10, Figure 11, Figure 13, and Figure 16) and figures showing the cutting gaps themselves (Figure 7, Figure 9, Figure 12, and Figure 14).
- The AWJ type waterjet produced a slightly wider cutting gap on the waterjet entry side into the material at all traverse speeds compared to the WJ type waterjet.
- The WJ showed very low machining efficiency for all materials tested. On the outlet side of the WJ from the materials, it was not possible to measure the width of the cutting gap due to heavy contamination of the gap edges by cut material burrs, which produced a very irregular shape of the cutting gap.
- In terms of statistical testing of the effect of the cutting head traverse speed on the width of the cutting gap, there was a statistically significant difference between the WJ technology at the waterjet inlet into the material and the AWJ technology at the waterjet inlet and outlet from the material. Thus, it was shown that the traverse speed of the cutting head had a significant effect on the width of the cutting gap or the taper angle.
- At a traverse speed of 50 mm·min^−1^, the taper angles of the cutting gap for all tested materials approached the optimum shape, i.e., a perpendicular cut. The PP + MOBIHEL material showed the most uniform cutting gap taper angle, 0°09′. The taper angle increased with increasing traverse speed. The only exception was the material PVC-U + POLURAN, where the taper angle decreased at a traverse speed of 1000 mm·min^−1^ compared to a traverse speed of 750 mm·min^−1^ (see Figure 17).
- The graphs in Figure 17 and Figure 18 show that specimens of the same base material with POLURAN coating showed a higher value of the taper angle of the cutting gap than specimens with MOBIHEL coating at all traverse speeds.
- SEM analysis did not show delamination of the POLURAN and MOBIHEL surface treatment layers from the base material PP and PVC-U. The cut surface was more uniform when using AWJ technology, showed no significant change in surface texture, and showed no change in the grooving process of the cut surface. The kerf width at the output using AWJ technology at a cutting head traverse speed of 50 mm·min^−1^ showed a regular straight cut, without significant deformation and burrs.

## Figures and Tables

**Table 1:** Variable technological parameters of the water jet.

Cut Number	1	2	3	4	5	6	7	8
Traverse speed (mm·min^−1^)	50	50	250	250	750	750	1000	1000
Waterjet type	WJ	AWJ	WJ	AWJ	WJ	AWJ	WJ	AWJ

**Table 2:** Statistical evaluation of the width of the cutting gap of polymer materials with surface treatment according to ANOVA F-test with the given parameter *p* at the significance level α = 0.05.

Kerf Width	Material Tested			
	PVC-U + MOBIHEL	PVC-U + POLURAN	PP + MOBIHEL	PP + POLURAN
AWJ inlet	0.0001	0.0001	0.0001	0.0001
AWJ outlet	0.0001	0.0001	0.0001	0.0001

**Table 3:** Calculated values of the taper angle of the cutting gap depending on the traverse speed of the waterjet cutting head.

Traverse Speed (mm·min^−1^)	Material Tested			
	PVC-U + MOBIHEL	PVC-U + POLURAN	PP + MOBIHEL	PP + POLURAN
50	0°67′	0°99′	0°09′	0°49′
250	1°79′	2°39′	1°44′	2°13′
750	2°99′	3°78′	2°95′	3°73′
1000	3°22′	3°59′		

## Data Availability

Data sharing is not available.
